# News and Notes

**Published:** 1905-10

**Authors:** 


### *NEWS AND NOTES.*

The Russian Red Cross has spent $11,000,000 in the course of the present war.

Dr. Stuart McGuire, Richmond, has been made president of the University College of Medicine.

Dr. James T. Wolfe, New Orleans, and Louis A. Mereaux, St. Bernard, were stricken by yellow fever August 30.

A report from Buenos Ayres states that six cases of plague were discovered recently in Tucman, Argentine Republic.

J. Y. Hicks, M.D., Tulane University, of Louisiana, New Orleans, 1852, died at his home in Moulton, Texas, aged 23.

The trustees of the State Sanitarium, Milledgeville, ask for an appropriation for 1906 of $394,000, and for 1907 of $407,000.

Previous to the application of any medicine it is necessary to remove any calculus which may be the primary cause of the inflammation.

Mr. Christopher Heath, F. R. C. S., the well-known surgeon and anatomist, and author of "Practical Anatomy," died recently in London.

THE health commissioner of Florida has warned the physicians of the State of the existence of dengue in Key West, Port Tampa and Tampa.

---

JOHN J. LAWRENCE, M.D., New York University, New York City, 1861, died at his home near Coakley, N. C., from dropsy, August 21, aged 68.

---

ELEVEN new cases of cholera have been reported from Manila. The total number of cases since the outbreak is reported as forty, with twenty-five deaths.

---

DR. AND MRS. W. S. GOLDSMITH and children have returned from the North, and will take possession next week of one of the Scott flats on Baker street.

---

E. H. YANCY, M.D., Macon, Ga., 1869, surgeon in the Confederate service during the Civil War, died at his home in Covington, Ga., July 1, aged 70.

---

DR. KOCH reaffirms that the microbe of sleeping sickness exists in the Tsetse fly in East Africa. He also cables that he has found a simple way to render the fly inocuous.

---

ALL the physicians of Trenton, N. J., have banded together in an effort to overcome lodge practice, and have signed an agreement not to accept contract lodge work in any form.

Horde Sharp, M. D., graduate of the University of Louisville Medical Department, 1872, died suddenly from heart disease at his home in Sharpsburg, Ky., August 19, aged 58.

---

Dr. Palmer Dudley, of New York, died recently in Liverpool from acute pulmonary tuberculosis, while en route for the International Medical Congress at St. Petersburg.

---

On August 9th the London County Council erected a tablet on the house in which Edward Jenner, the discoverer of vaccination, lived in 1803. The tablet is of green encaustic ware.

---

The Paris Académie des Sciences Morales et Politiques has awarded to M. Vallery-Radot the Corday prize for his life of his father-in-law, Louis Pasteur. The prize consists in an annuity of $50.

---

It is reported that, after a period of quiescence, plague has again made its appearance in Tokio, Japan, and that the disease seems to be spreading in spite of the rigid quarantine which is enforced.

---

The State Department is in receipt of information from the Russian government that the meeting of the Fifth International Congress on Obstetrics and Gynecology has been postponed for one year.

---

The president of Nicaragua has issued a proclamation declaring the ports of that country closed to all vessels from Panama because of reports of the presence of bubonic plague in the latter republic.

THE Piedmont Sanatorium has been opened under the charge of Dr. Ludwig Amster, with Dr. Maloney, formerly of the Grady Hospital, as assistant. Dr. Floyd McRae will have charge of the surgical department.

---

MILK coming from a large dairy farm in Pennsylvania was found to have a pink tinge which increased in tint on standing. Investigation resulted in the isolation of one cow from the herd, and the proving that the coloring-matter was due to the Bacillus Prodigious.

---

ALAN GREEN, bacteriologist in charge of the vaccine lymph department, Lister Institute of Preventive Medicine, has been awarded a silver medal by the French Academy of Medicine for his work on vaccine, published in the proceedings of the Royal Society.

---

THE town council of Moscow, says the *St. Petersburger Medicinische Wochenschrift*, has decided to erect at once an asylum for epileptics, for which a special fund of 700,000 rubles is at the council's disposition. The first instalment of the building will accommodate 150 patients.

---

IT is announced that the first American Tuberculosis Exhibition will be held in New York next November under the auspices of the National Association for the Study and Prevention of Tuberculosis and of the committee on Prevention of Tuberculosis of the Charity Organization Society.

DR. SENN sailed on July 17th from Cape Sabine, North Labrador, on a vessel which is going north with supplies for the Peary expedition. It is said Dr. Senn will not attempt to reach the North Pole, but he desires to have some experience in Arctic travel with the Peary expedition.

---

THE legislature of the State of California has passed a law making it a misdemeanor to own, to lease, to let, or to hire any room for a lodging or sleeping apartment that contains less than 500 cubic feet of air space "in the clear" for each occupant; it is also a misdemeanor to be an occupant of such apartment.

---

THE authorities in the duchy of Hesse, Germany, have notified druggists throughout the country that when a physician's prescription is written illegibly—as frequently happens—the druggist is not to waste any time guessing at the writer's meaning, but to require from the physician a legible copy or indication of the contents.

---

THE fifteenth annual meeting of the New York and New England Association of Railway Surgeons will be held at the Academy of Medicine, New York City, November 17-18, 1905, under the presidency of Dr. G. P. Conn, of Concord, N. H. One-half day of the meeting will be devoted to a symposium on "Injuries to the Head and Spine."

---

DR. THOMAS MENEES died at his home near Nashville, Tenn., September 7th, aged 83. In 1861 Dr. Menees was elected a member of the Confederate Congress and remained a member of that body until the dissolution of the Confederate government. The deceased had been professor of obstetrics in the University of Nashville and in Vanderbilt University.

The Congress of the International Society of Surgery will hold its first meeting this year at Brussels, under the patronage of His Majesty Leopold II., King of the Belgians. The congress will be in session from Monday, September 18, to the following Saturday inclusive, under the presidency of Theodor Kocher, M. D., professor of surgery in the University of Berne.

---

Dr. G. C. M. Godfrey, assistant surgeon United States army, committed suicide September 23d, at Fort McPherson.

Dr. Godfrey was the son of Colonel E. S. Godfrey, commanding officer of the 9th cavalry, now stationed at Fort Riley, Kansas.

The general belief among the officers at Fort McPherson is that the cause of the suicide was temporary mental aberration.

---

The Hall County Medical Society has been organized by Dr. L. G. Hardman, organizer for the State Association for the ninth congressional district. The following were elected officers: President, J. W. Bailey; vice-president, J. W. Oslin; secretary and treasurer, P. E. B. Robertson; board of censors, J. H. Downey, J. B. Rudolph and W. A. Palmour. There were sixteen physicians to join at the first meeting. The society will meet again the first Tuesday in October.

---

As a result of a fire the University of Pennsylvania has sustained a loss of $40,000. The damaged buildings are in such condition, however, that repairs can easily be effected; at first it was thought that the microscopes and other scientific apparatus were destroyed, but investigation showed that the anatomical and surgical preparations which were lost can be replaced without much difficulty. The cause of the fire is not known, but crossed electric wires are thought to have given birth to the flames.

